# Identification of a Recurrent *STRN/ALK* Fusion in Thyroid Carcinomas

**DOI:** 10.1371/journal.pone.0087170

**Published:** 2014-01-27

**Authors:** Gaëlle Pérot, Isabelle Soubeyran, Agnès Ribeiro, Benjamin Bonhomme, Frédérique Savagner, Nathalie Boutet-Bouzamondo, Isabelle Hostein, Françoise Bonichon, Yann Godbert, Frédéric Chibon

**Affiliations:** 1 Department of Biopathology, Institut Bergonié, Bordeaux, France; 2 INSERM U916, Institut Bergonié, Bordeaux, France; 3 INSERM U694, Institut Biologie Santé, Angers, France; 4 Department of Nuclear Medicine, Institut Bergonié, Bordeaux, France; 5 Translational Research, Institut Bergonié, Bordeaux, France; Ohio State University Medical Center, United States of America

## Abstract

Thyroid carcinoma is the most common endocrine malignant tumor and accounts for 1% of all new malignant diseases. Among all types and subtypes of thyroid cancers that have been described so far, papillary thyroid carcinoma is the most frequent. The standard management treatment of these tumors consists of surgery, followed by radioiodine treatment in case of high risk of relapse. The most aggressive forms are commonly treated by chemotherapy, radiotherapy or experimental drug testing. We recently reported the case of a patient presenting an anaplastic thyroid carcinoma with lung metastases. Fluorescence *in situ* hybridization analysis allowed us to detect a rearrangement of the anaplastic lymphoma kinase (*ALK*) gene in both tumors. The patient was treated with crizotinib and presented an excellent drug response. We present here the subsequent investigations carried out to further characterize this genetic alteration and to assess the prevalence of *ALK* rearrangements in thyroid lesions. High resolution array-comparative genomic hybridization data complemented by RT-PCR and sequencing analyses, allowed us to demonstrate the presence of a *STRN/ALK* fusion. The *STRN/ALK* transcript consisted of the fusion between exon 3 of *STRN* and exon 20 of *ALK*. Subsequent screening of 75 various thyroid tumors by RT-PCR revealed that 2 out of 29 papillary thyroid carcinomas exhibited the same fusion transcript. None was detected in other types of malignant or benign thyroid lesions analyzed. These findings could pave the way for the development of new targeted therapeutic strategies in the treatment of papillary thyroid carcinomas and point to ALK inhibitors as promising agents that merit rapid evaluation.

## Introduction

Thyroid carcinoma is the most common malignancy of the endocrine system and accounts for 1% of all new malignant diseases. Among the different thyroid cancer types described, papillary thyroid cancer (PTC) and follicular thyroid cancer (FTC) are the most frequent, accounting for 80–85% and 10–15% of thyroid malignancies, respectively [1]–[2].

PTC is a well-differentiated tumor defined by its characteristic aspect of the cell nuclei. Fifteen histological variants have been described in the World Health Organization (WHO) classification [1]. The prognosis for patients with PTC is good, with the 10-years survival rate being over 93% [3]–[4]. Some histological variants, as well as the presence of a poorly differentiated component, tend to show a more aggressive clinical behaviour.

FTC is a well-differentiated hypercellular tumor presenting a follicular differentiation pattern and lacking the nuclear features of PTC [1]–[2]. Follicular carcinomas are usually encapsulated and can be minimally to widely invasive. The 5-year survival rate is 80 to 98% for minimally invasive FTC and 38% for widely invasive tumors [5].

Anaplastic thyroid carcinoma (ATC) is a rare (1–2%) but very aggressive form of thyroid cancer. This highly malignant tumor is composed of undifferentiated cells along with necrosis and mitosis. ATC can be a *de novo* tumor or derive from PTC or FTC [2].

The standard management treatment for both PTC and FTC tumors is surgery followed by radioiodine if the risk of relapse is high or very high. In some cases, despite favorable outcomes late relapses are possible, and patients might die after local recurrence or metastases [6]. Moreover, about 10% of patients will experience advanced radioactive iodine refractory cancer with a poor prognosis and low response to chemotherapy [7]–[8]. Nevertheless, encouraging results have been obtained with the use of kinase inhibitors in the context of prospective trials [9]. In the case of the very malignant anaplastic thyroid carcinoma, with less than 20% of the patients surviving one year [10], there is urgent need for new therapeutic approaches. Although a multimodal approach was recently reported as being beneficial by Smallridge *et al*. [11], the elucidation of molecular events underlying carcinogenesis and the development of kinase inhibitors as potential therapeutic anticancer agents are emerging as promising strategies for the treatment of this malignant disease.

We recently reported the case of a 71-year-old woman presenting an anaplastic thyroid carcinoma with PTC component and lung metastases. A rearrangement of the anaplastic lymphoma kinase (*ALK*) gene was detected using FISH [12] both in the well-differentiated and anaplastic components of the thyroid tumor and in the anaplastic lung metastases. The patient was treated with crizotinib and presented an excellent response of >90% across all pulmonary lesions (criteria RECIST 1.1) that was confirmed at 6 months after therapy initiation [12]. These remarkable results provide new therapeutic options for these very poor carcinomas and potentially for all carcinomas presenting an *ALK* rearrangement.

We report here the subsequent identification of *STRN* gene as the *ALK* fusion partner and the screening via RT-PCR of a series of 75 thyroid carcinoma samples in order to test the presence of the *STRN/ALK* fusion.

## Materials and Methods

### Ethics statement

The samples used for this study were provided by the Biological Resources Centers of Institut Bergonié (CRB-IB) and the Academic Hospital of Angers (CRB-A) and were anonymized prior to research.

The samples were centralized in the Biological Resources Center of Institut Bergonié (http://intranet.bergonie.org/DDS/biopathologie/SitePages/Centre%20de%20ressources%20biologiques.aspx?WikiPageMode=Edit&InitialTabId=Ribbon.EditingTools.CPEditTab&VisibilityContext=WSSWikiPage), which received the agreement from the French authorities (French Public Health Code articles L. 1243-4 and R. 1243-61) to deliver samples for scientific research (number AC-2008-812, on February 2011), approved by the Committee of Protection of Individuals. The project was approved by the Bergonié ethic committee (scientific advisory board).

### Tumor samples and histological features

Tumor diagnosis was performed according to the World Health Organization classification [1]. The first tumors explored in this study consisted of an anaplastic thyroid carcinoma (ATC) with PTC component and its lung metastases (for case description see reference 12). FFPE material was available for both localizations. Normal thyroid tissue was also used in this study.

The screened series was composed of 75 thyroid tumors: 32 were papillary carcinomas (PTC), 11 follicular carcinomas (FTC), 2 poorly differentiated carcinomas (PDTC), 1 PDTC with a PTC component, 2 papillary type PDTC, 5 oncocytic carcinoma (OTC) and 25 benign tumors (Table S1). Among PTC, 16 corresponded to the classic type, 11 to a follicular variant, 1 to a solid variant and 1 to an oncocytic variant. Among FTC, 3 corresponded to the classic type and 8 to minimally invasive FTC. One FTC specimen corresponded to metastasis. Among benign tumors, 10 corresponded to follicular adenoma (FTA) and 15 to oncocytic adenoma (OTA). In 61 cases, frozen tissue was available and in the other 14 cases only fixed blocks of paraffin were available (Table S1).

For PCR control C1 sample, which corresponds to an Ewing-like sarcoma with a *CIC-DUX4* translocation, was used.

### DNA extraction and array-CGH

Genomic DNA was extracted according to Agilent protocol for DNA isolation on FFPE tissue (http://www.chem-agilent.com/pdf/G4410-90020v3_1_CGH_ULS_Protocol.pdf) (Agilent Technologies) and array-CGH experiment was performed as previously described using SurePrint G3 Human CGH Microarray Kit, 1×1 M (Agilent Technologies) [13].

### RNA extraction and RT-PCR

RNA extraction from frozen or FFPE tissue and reverse transcription using random hexamers (RT) were performed as previously described [13]. A specific reverse transcription was also performed on samples using a reverse *ALK* primer (*ALK*ex20R1) (Table S2). For PCR, primers used were designed using the Primer 3 program (http://frodo.wi.mit.edu/primer3/) and are presented in Table S2. Control PCR were performed using *STRN* forward and reverse primers or *ALK* forward and reverse primers.

For fusion transcript detection, PCR were performed, on both classical RT and *ALK* specific RT, with different *STRN* forward primers and the *ALK* reverse primer: *ALK*ex20R2 (Table S2). For FFPE samples only potential fusion between exons 3 to 7 of *STRN* with *ALK* exon 20 were screened because of the low quantity of RNA obtained. For frozen samples fusions between exons 3 to 18 of *STRN* and *ALK* exon 20 were screened (Table S2). PCR were performed on 50 ng of cDNA using AmpliTaqGold® DNA polymerase (Applied Biosystems) with an annealing temperature of 60°C.

### Genomic DNA extraction and PCR

Genomic DNA from a frozen sample was isolated using a standard phenol-chloroform extraction protocol. For PCR, primers were designed using the Primer 3 program (http://frodo.wi.mit.edu/primer3/) and are presented in Table S3. All combinations of PCR were performed as previously described for RT-PCR on 50 ng of gDNA using AmpliTaqGold® DNA polymerase (Applied Biosystems) with an annealing temperature of 60°C.

### Sequencing

PCR products were purified using an ExoSAP-IT PCR Purification Kit (GE Healthcare) and sequencing reactions were performed with the Big Dye Terminator V1.1 Kit (Applied Biosystems) according to the manufacturer's recommendations. Samples were purified using the Big Dye XTerminator Purification kit (Applied Biosystems) according to the manufacturer's instructions and sequencing was performed on a 3130xl Genetic Analyzer (Applied Biosystems).

### Immunohistochemistry

Immunohistochemistry was performed on formalin-fixed paraffin embedded 4 µm-thick tissues on a BenchMark Ultra instrument (Ventana). The primary antibody and dilution used in this study are as follows: rabbit monoclonal anti-ALK (clone D5F3; Cell Signaling Technology, Danvers, MA) applied at 1:50 and detected with Ultraview Universal DAB detection kit (Ventana). IHC pictures were taken using a Leitz DMRB microscope (Leica) and a DS-Ri1 camera (Nikon).

### Fluorescence *in situ* hybridization

FISH assay was performed using the Histology FISH accessory kit (Dako) as previously described according to the manufacturers' instructions [14]. Interphase molecular cytogenetic studies using a commercially-available *ALK* probe (Vysis LSI ALK Dual Color, Break Apart Rearrangement Probe, Abbott Molecular) were performed on a 4-µm paraffin-embedded section. Nuclei were scored for non-rearranged patterns (red and green signals overlapping or close together) and unbalanced patterns (split red and green signals or single red signals) using a Nikon Eclipse 80i fluorescent microscope with appropriate filters. Pictures were captured using a Hamamatsu C4742-95 CCD camera and analyzed with the Genikon software (Alphelys). The positive threshold was defined as more than 15% of signals split and/or isolated red signal as previously described [15] in 100 tumor cells.

## Results

### *STRN/ALK* fusion identification in an anaplastic thyroid carcinoma and in its lung metastases

**Figure 1:**
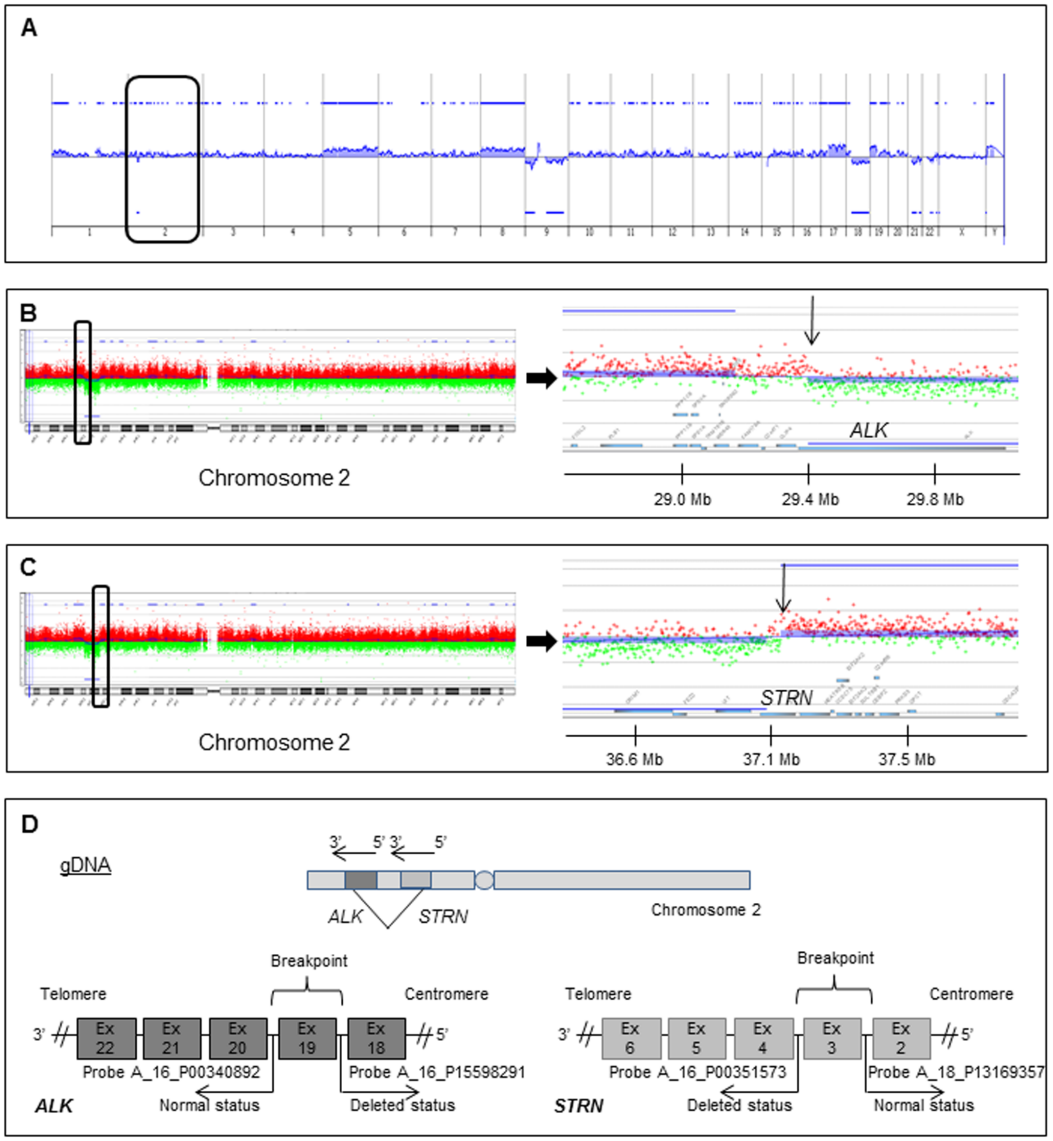
Genomic profile. (A) CGH profile obtained from a pulmonary metastasis of the initial case. Genomic alterations are presented and organized on the X axis from chromosome 1 to 22 and X, Y. Log2 ratio values are reported on the Y axis. Significant gains or losses are indicated by blue lines and blue areas above or below each profile, respectively. Chromosome 2 is highlighted with a black box. (B) Enlargements of chromosome 2 and *ALK* chromosomal region. The chromosome 2 region containing *ALK* locus is highlighted with a black box. The log2 ratio values of probes covering the proximal regions of *ALK* and the gene itself are presented. The arrow indicates the breakage region. (C) Enlargements of chromosome 2 and *STRN* chromosomal region. The chromosome 2 region containing *STRN* locus is highlighted with a black box. The log2 ratio values of probes covering the proximal regions of *STRN* and the gene itself are presented. The arrow indicates the breakage region. (D) Schematic representation of potential breakpoints into the two genes at the genomic level (gDNA) according to the CGH data. Agilent CGH probes surrounding the different breakpoints are indicated.

We have recently reported a case of an *ALK* gene rearrangement detected by FISH both in an anaplastic thyroid carcinoma (ATC) with PTC component and in its lung metastases [12]. FISH revealed that 52% of primary thyroid tumor cells and 66% of lung metastasis carcinoma cells presented an unbalanced rearrangement of the *ALK* gene. The observation of this unbalanced rearrangement prompted us to perform array-CGH experiments. Previous array-CGH results, showed a deletion on chromosome 2 starting in the *ALK* gene and expanding from the region 2p23.2 to the region 2p22, in both localisations [12]. However the low CGH resolution did not allow us to perform a fine mapping of the breakpoints. For this purpose a new array-CGH experiment with a higher resolution was performed for the lung anaplastic carcinoma with adequate DNA quality and quantity. The tumor genomic profile displayed gains of the chromosomes 5, 8, 17q, 19p and losses of chromosomes 9, 18q, 21 and 22 (Figure 1A). A deletion of approximately 7.7 Mb from the 2p23.2 region to the 2p22.2 could also be observed (Figures 1A and B). A more detailed look at the deletion extremities on chromosome 2 revealed a breakpoint in the 3′ end of the *ALK* gene (Figure 1B). Most of the gene was lost whereas the last 3′ probes in *ALK* and the 3′ probes covering the adjacent *ALK* region were not deleted, these results being consistent with those obtained by FISH. The chromosome 2 deletion extended until the 5′ end of the *Striatin* gene (*STRN*) (Figure 1C). Since both genes (*ALK* and *STRN*) are oriented in the same direction on the same chromosome; we hypothesized that the deletion of the region between the two genes could lead to the fusion of the 5′ part of the *STRN* gene with the 3′ part of the *ALK* gene.

The log2 ratio values of CGH probes covering *ALK* and *STRN* regions allowed us to see that the deletion breakpoints were situated in *ALK* between the Agilent probes A_16_P00340892 located in intron 19 and A_16_P15598291 situated in intron 18 and *STRN* gene between the probes A_16_P00351573 in intron 3 and A_18_P13169357 located in intron 2 (Figure 1D).

**Figure 2:**
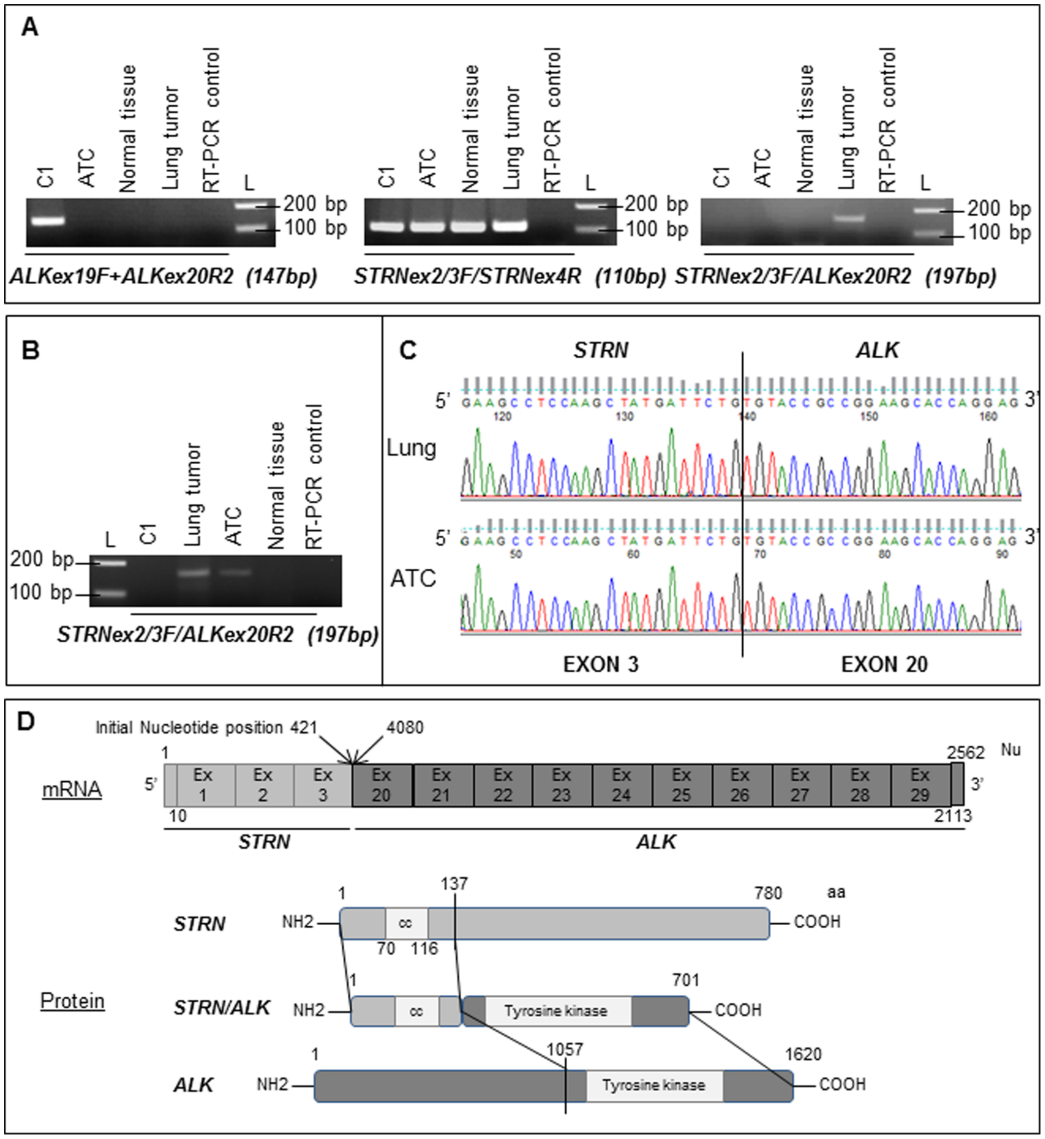
*ALK*, *STRN* and *STRN/ALK* fusion transcript RNA expression and sequencing. (A) Expression of *ALK*, *STRN* and *STRN/ALK* fusion transcript obtained by RT-PCR in the pulmonary tumor, the thyroid tumor and non tumoral tissue of the initial sample, and in one control sample (C1) are presented. L: molecular weight ladder, bp: base-pair. (B) Expression of *STRN/ALK* fusion transcript obtained by an *ALK* specific RT using *ALK*ex20R1 primer followed by PCR in the same samples. (C) Chromatogram showing the sequence of *STRN/ALK* fusion transcript at the breakpoint observed in the pulmonary and thyroid carcinomas. The *STRN* exon 3 (NM_003162) at the 5′ part of the transcript is fused to the *ALK* exon 20 (NM_004304) at the 3′ portion. (D) Schematic representation of the structure of the fusion transcript, of ALK and STRN proteins and of the predicted fusion protein. For the fusion transcript, initial position of the nucleotides at the fusion are indicated (position on *STRN* and *ALK* mRNA). Nu: nucleotide, aa: amino acid, cc: coiled-coil domain. Ensembl genome browser ID: *ALK* (gene: ENSG00000171094, mRNA: ENST00000389048, prot: ENSP00000373700) *STRN* (gene: ENSG00000115808, mRNA: ENST00000263918, prot: ENSP00000263918).

In order to prove that the *STRN* gene was the *ALK* partner, RT-PCR was performed on paraffin-embedded tumors (Table S2). First we could observe that only C1 sample was positive for *ALK* expression and that *STRN* was well-expressed in all studied samples (Figure 2A). Secondly regarding the *STRN/ALK* RT-PCR, a potential fusion RT-PCR product could only be obtained from the positive lung sample (Figure 2A). Therefore, in order to detect the fusion in the ATC tumor, we performed an *ALK* specific reverse transcription using an *ALK* reverse primer followed by PCR. A potential fusion transcript was detected both in ATC and in lung metastases (Figure 2B). No RT-PCR product was observed neither in the control sample C1 nor in the normal thyroid tissue for the two experiments (Figures 2A and 2B). Sequencing of the RT-PCR products (ATC and lung samples) confirmed a fusion between exon 3 of *STRN* and exon 20 of *ALK* in both cases (Figure 2C). The in-frame fusion transcript sequence was expected to contain 2562 nucleotides (including UTR regions) and the predicted protein to exhibit 701 amino acid residues (Figure 2D).

### *STRN/ALK* fusion screening in several histotypes of thyroid tumors

**Figure 3:**
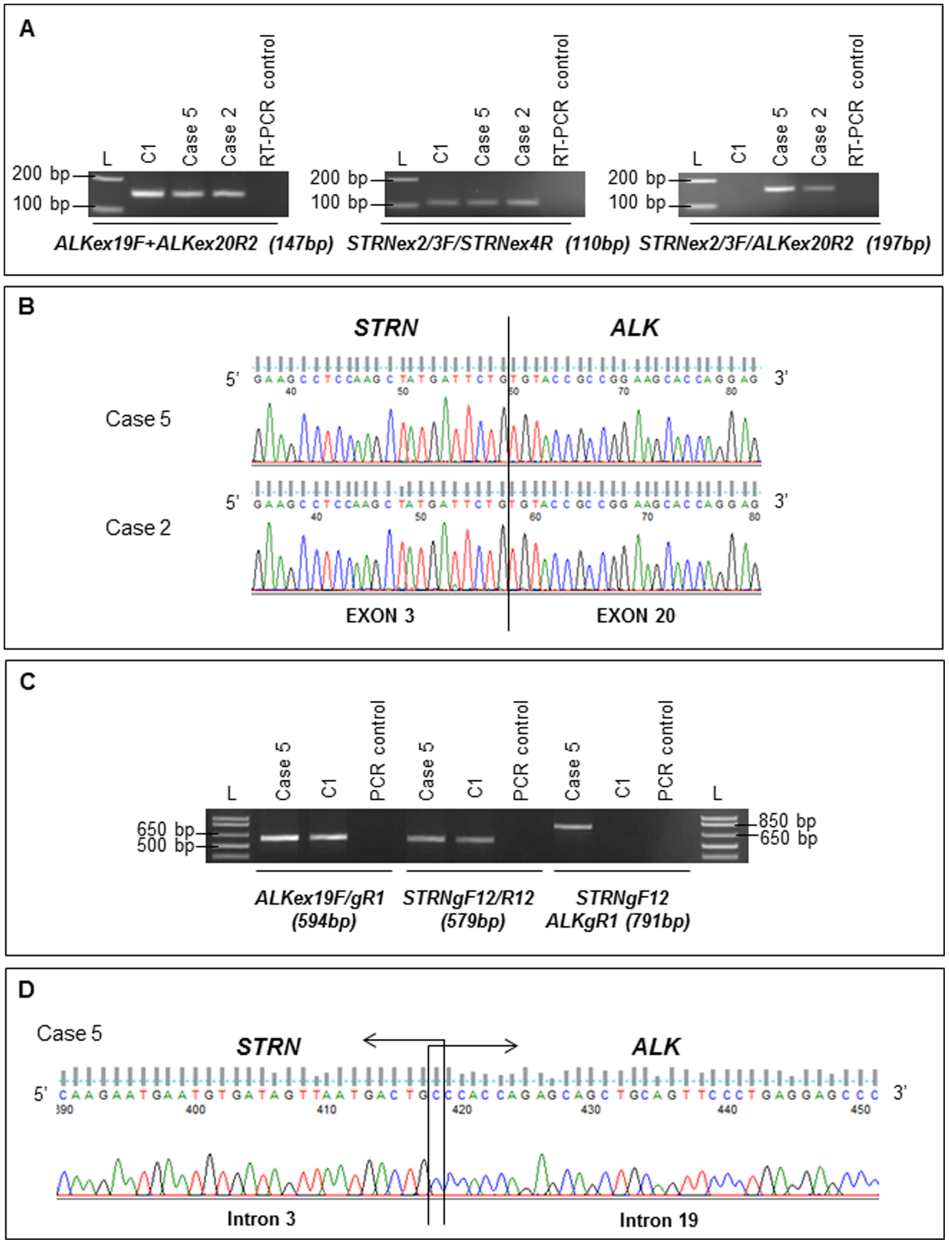
*ALK*, *STRN* and *STRN/ALK* fusion status at mRNA and genomic DNA levels in positive PTC. (A) Expression profiles of *ALK*, *STRN* and *STRN/ALK* fusion transcript obtained by RT-PCR in two PTC samples and in one control sample (C1) are presented. L: molecular weight ladder, bp: base-pair. (B) Chromatogram showing the sequence of *STRN/ALK* fusion transcript at the breakpoint observed in the two PTC samples. The *STRN* exon 3 (NM_003162) at the 5′ part of the transcript is fused to the *ALK* exon 20 (NM_004304) at the 3′ portion. (C) Products obtained after PCR on genomic DNA using first *ALK* and *STRN* forward and reverse primers and then the combination of a *STRN* forward primer with an *ALK* reverse primer for case 5 and one control sample (C1). (D) Chromatogram showing the sequence of *STRN/ALK* fusion at the genomic breakpoint observed in the case 5. One nucleotide at the intronic junction (C) is identical in both fused genes and might be contributed by either of them.

In order to test whether the *STRN/ALK* fusion is recurrent in thyroid tumors, 75 thyroid samples of various histotypes were screened by RT-PCR (Tables S1 and S2). Expression of the *STRN/ALK* fusion transcript was detected in two tumors (cases 2 and 5) among the 75 (Figure 3A). Both cases presented the nucleotidic sequence previously observed in the ATC and lung metastases with the fusion between exon 3 of *STRN* and exon 20 of *ALK* (Figure 3B).

As frozen material was available for case 5, we used PCR to search for the *STRN*/*ALK* fusion at the genomic level. A PCR product which measured 791 bp was obtained (Figure 3C) and was constituted of 441 bp of *STRN* intron 3 at its 5′end fused to 349 bp of *ALK* intron 19 at its 3′end (Figure 3D). One nucleotide, a cytosine, located at the junction overlapped in the sequences of both genes and might originate from either *STRN* or *ALK*. No PCR products were detected screening the two other positive cases with these primers and other primers closer to breakpoints (data not shown).

**Figure 4:**
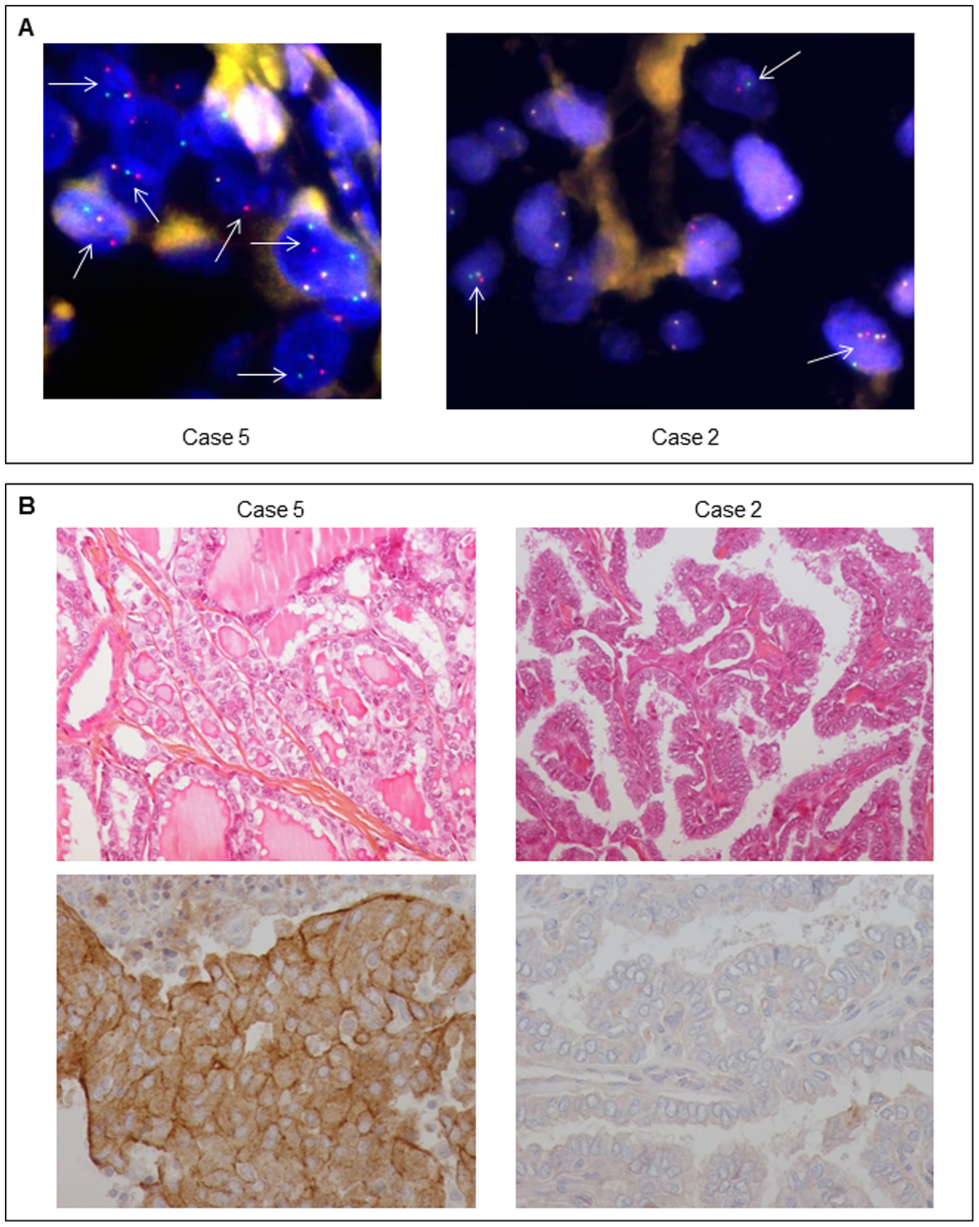
ALK Interphase fluorescence *in situ* hybridization and immunohistochemistry. (A) On the representative picture of the FISH, realized using the LSI ALK Dual Color Break Apart Rearrangement Probe, we could observe *ALK* probe break-aparts highlighted by arrows in the two positive PTC cases. Magnification: X1000. (B) Pictures of immunohistochemical labeling for ALK in the two PTC samples. Pictures of hematoxylin, eosin and safran (HES) staining of the two samples are also presented. Magnification for HES: X200, Magnification for IHC: X400.

FISH was subsequently performed to confirm the presence of an *ALK* rearrangement in these two cases (Figure 4A). Case 5 displayed 62% of cells with one fusion signal and separate green and red signals, whereas 20% showed one fusion signal and a red signal only. Case 2 exhibited 20% of cells with one or more fusion signals with separate green and red signals. It is widely accepted that both patterns (*i.e.* cells with one fusion signal and separate green and red signals, and cells with one fusion signal and a red signal only) correspond to rearranged *ALK* profiles.

ALK immunohistochemistry was also performed and we found an ALK overexpression in case 5 but none in case 2 (Figure 4B).

### Occurrence of *STRN/ALK* fusion in thyroid cancers

The two positive cases for *STRN/ALK* fusion were identified in conventional PTC (Table S1) revealing that 2/16 of conventional PTC harbor a rearrangement between *STRN* and *ALK*. The fusion was not observed in PTC variant forms (13 cases), in follicular thyroid carcinomas (11 cases), in poorly differentiated carcinomas (5 cases), in oncocytic thyroid carcinomas (5 cases) or in benign lesions including follicular thyroid adenoma (10 cases) and oncocytic thyroid adenoma (15 cases).

## Discussion

The classification of thyroid carcinoma into different histotypes is essentially based on morphological and clinical features [1]–[2] and advances in molecular studies of these tumors have confirmed the relevance of this pre-existing classification. In PTC, the most frequent genetic alterations are the translocation of receptor tyrosine kinase genes *RET* (20 to 30%) and *TRK* (10%), point mutations of *RAS* family genes and *BRAF* mutations [1]–[2]. These alterations are generally mutually exclusive in PTC [2].

The present study was initiated following the detection of an *ALK* rearrangement in an ATC with PTC component and its lung metastases [12]. To date, there is only one publication on the presence of rearranged *ALK* gene in PTC [16]. Hamatani et al. described *ALK* rearrangement in 52.6% (10/19) of cases of a radiation exposed-patient cohort [16]. In six positive cases, *EML4* was found as the *ALK* fusion partner whereas in the other four the *ALK* partner has not been identified yet. No rearrangement was observed in PTC patients with no radiation exposure.

High resolution array-CGH analysis and RT-PCR followed by sequencing allowed us to identify a fusion between the *STRN* exon 3 and the *ALK* exon 20 in the metastatic lung tumor. The same fusion was identified in the ATC primary tumor. *STRN* gene encodes the striatin, a calmodulin-binding member of the WD repeat family of proteins, presenting a coiled-coil domain [17]. The anaplastic lymphoma kinase (ALK) is a protein tyrosine kinase from the insulin receptor subfamily [18]. The *ALK* gene was initially identified as a fusion partner of nucleophosmin in anaplastic large-cell lymphoma [19]–[20]. The *STRN/ALK* fusion observed by us may encode a predicted protein consisting of the fusion of the N-terminal part of the STRN protein, retaining its coiled-coil domain with the C-terminal part of ALK protein, including its tyrosine kinase domain. Although *ALK* has been described to be fused to many different partners [18], the shared feature of all translocation products is the conservation of the ALK tyrosine kinase domain that is fused to a part of a protein displaying a coiled-coil domain [21]–[22].

To date, only two publications have described the implication of *STRN* in a translocation: in a chronic eosinophilic leukaemia case (CEL) [23] and in a non-small cell lung carcinoma (NSCLC) [24]. In the CEL case, the authors identified a fusion between the *STRN* exon 6 and a truncated *PDGFRA* exon 12. Similarly to the *ALK* gene, *PDGFRA* encodes a tyrosine kinase receptor and the predicted fusion protein retains both the coiled-coil domain of STRN and the tyrosine kinase domain of the receptor. Even though the STRN part implicated in the CEL case is larger than the one we report, the coiled-coil domain is conserved in both cases [23]. In the NSCLC case the *STRN/ALK* fusion resulted in the fusion of *STRN* exon 3 with the *ALK* exon 20, with the same nucleotidic sequence observed in our cases [24].

In order to see if the *STRN/ALK* fusion transcript could be observed in other thyroid carcinomas we have performed RT-PCR screening on 75 thyroid tumors (50 malignant and 25 benign). Two conventional PTC with a *STRN/ALK* fusion could be identified from the screening. Both of them presented the same sequence as the first ATC case and the previously described NSCLC [24]. It seems that in PTC and in NSCLC, the fusion occurs preferentially between the *STRN* intron 3 and the *ALK* intron 19. The precise DNA breakpoints were obtained for only one sample (case 5) and remain to be determined in other cases.

To independently confirm the presence of an *ALK* rearrangement in these two cases, FISH experiment using an *ALK* Break-apart probe was carried out. Interestingly distinct rearrangement patterns could be observed by FISH in these thyroid tumors. We could hypothesize that in the case of cells with one fusion signal and one red signal (no green signal, as in the described ATC case and its metastases, [12]) the fusion of the two genes could be due to a deletion of the chromosome 2 region between the two genes, while the pattern of one fusion signal with separate green and red signals (cases 2 and 5) could correspond to a translocation occurring between the two homologous chromosomes 2.

Concerning immunohistochemical (IHC) results, among the three *ALK* rearranged cases, case 2 was the only one not to show ALK positive staining, even if IHC and FISH were performed on the same tumor area (for PTC first case with lung metastases IHC results see reference [12]). ALK expression in *ALK* rearranged tumors is usually detectable by immunohistochemistry using ALK D5F3 clone, however false negative results might be obtained in some cases [25]–[27]. Also the fixator used was not formalin but “Holland Bouin” which could explain a false negative result. For this case we could not formally conclude if the absence of immunostaining corresponded to a false negative result or to a lack of protein expression, even if the latter is unlikely.

Morphologically, the two *ALK*-rearranged cases (cases 2 and 5) corresponded to conventional PTC. We did not observe either an anaplastic component as we did in the case previously reported, or a solid/trabecular like architecture as observed by Hamatani *et al*. in 60% of their positive cases [16]. However these two cases showed aggressive features with lymph nodes metastases at diagnosis.

The 75 thyroid tumors series study revealed that 2 among 16 conventional PTC exhibited a *STRN*/*ALK* fusion. Additionally, the fusion seems to be unique to PTC in this series since no transcript was detected in other malignant thyroid cases (11 FTC, 5 PDTC and 5 OTC) or in benign thyroid lesions (10 FTA and 15 OTA). However, the study was not representative of the thyroid carcinoma incidence. In order to assess the prevalence of *ALK*-rearrangements, and particularly the *STRN/ALK* fusion occurrence in thyroid tumors more precisely, a larger batch of papillary thyroid carcinomas and anaplastic thyroid carcinomas will be screened *via* FISH and RT-PCR in the near future.

To conclude, this study offers new perspectives on the treatment of papillary thyroid carcinomas, especially in radioiodine refractory patients. The search of *ALK* rearrangements in thyroid cancers may be as important as in NSCLC and the efficiency of treatment by ALK inhibitors must be further evaluated.

## Supporting Information

Table S1 **Sample description.** Histotype, material available and RT-PCR results for each studied case are presented. PTC: papillary thyroid carcinoma, FV-PTC: follicular variant-PTC, FTC: follicular thyroid carcinoma, Min inv FTC: minimally invasive FTC, FTA: follicular thyroid adenoma, OTA: oncocytic thyroid adenoma, OTC: oncocytic thyroid carcinoma, FFPE: Formalin-Fixed Paraffin Embedded, HBFPE, Holland Bouin Fixed Paraffin Embedded.
(DOC)

Table S2 **RT-PCR primers used.** *ALK* and *STRN* forward and reverse primers are presented. Ex: exon, bp: base pair, FFPE: Formalin-fixed paraffin embedded, F: forward, R: reverse.
(DOC)

Table S3 **Genomic DNA primers used.** *ALK* and *STRN* forward and reverse primers are presented. Ex: exon, bp: base pair.
(DOC)
