# Supplementary material for: Identification of a Recurrent STRN/ALK Fusion in Thyroid Carcinomas
Source: PLoS One. 2014 Jan 27;9(1):e87170. doi: 10.1371/journal.pone.0087170 (PMC3903624; doi:10.1371/journal.pone.0087170)
Supplement: Table S1 — Sample description. Histotype, material available and RT-PCR results for each studied case are presented. PTC: papillary thyroid carcinoma, FV-PTC: follicular variant-PTC, FTC: follicular thyroid carcinoma, Min inv FTC: minimally invasive FTC, FTA: follicular thyroid adenoma, OTA: oncocytic thyroid adenoma, OTC: oncocytic thyroid carcinoma, FFPE: Formalin-Fixed Paraffin Embedded, HBFPE, Holland Bouin Fixed Paraffin Embedded. (DOC) [file pone.0087170.s001.doc]

| **Cases** | **Histotype** | **Material** | **RT-PCR** | **Cases** | **Histotype** | **Material** | **RT-PCR** |
| --- | --- | --- | --- | --- | --- | --- | --- |
| **1** | PTC | Frozen | Negative | **38** | Min inv FTC | Frozen | Negative |
| **2** | PTC | HBFPE | **Positive** | **39** | Min inv FTC | Frozen | Negative |
| **3** | PTC | Frozen | Negative | **40** | Min inv FTC | Frozen | Negative |
| **4** | PTC | FFPE | Negative | **41** | Min inv FTC | Frozen | Negative |
| **5** | PTC | Frozen | **Positive** | **42** | Min inv FTC | Frozen | Negative |
| **6** | PTC | FFPE | Negative | **43** | Min inv FTC | Frozen | Negative |
| **7** | PTC | Frozen | Negative | **44** | Min inv FTC | Frozen | Negative |
| **8** | PTC | Frozen | Negative | **45** | Min inv FTC | Frozen | Negative |
| **9** | PTC | Frozen | Negative | **46** | FTA | Frozen | Negative |
| **10** | PTC | Frozen | Negative | **47** | FTA | Frozen | Negative |
| **11** | PTC | Frozen | Negative | **48** | FTA | Frozen | Negative |
| **12** | PTC | Frozen | Negative | **49** | FTA | Frozen | Negative |
| **13** | PTC | Frozen | Negative | **50** | FTA | Frozen | Negative |
| **14** | PTC | Frozen | Negative | **51** | FTA | Frozen | Negative |
| **15** | PTC | Frozen | Negative | **52** | FTA | Frozen | Negative |
| **16** | PTC | Frozen | Negative | **53** | FTA | Frozen | Negative |
| **17** | PDTC with PTC component | FFPE | Negative | **54** | FTA | Frozen | Negative |
| **18** | PTC solid variant | Frozen | Negative | **55** | FTA | Frozen | Negative |
| **19** | PDTC papillary type | FFPE | Negative | **56** | OTA | Frozen | Negative |
| **20** | PDTC papillary type | FFPE | Negative | **57** | OTA | Frozen | Negative |
| **21** | Oncocytic PTC | FFPE | Negative | **58** | OTA | Frozen | Negative |
| **22** | FV-PTC | Frozen | Negative | **59** | OTA | Frozen | Negative |
| **23** | FV-PTC | FFPE | Negative | **60** | OTA | Frozen | Negative |
| **24** | FV-PTC | FFPE | Negative | **61** | OTA | Frozen | Negative |
| **25** | FV-PTC | FFPE | Negative | **62** | OTA | Frozen | Negative |
| **26** | FV-PTC | FFPE | Negative | **63** | OTA | Frozen | Negative |
| **27** | FV-PTC | FFPE | Negative | **64** | OTA | Frozen | Negative |
| **28** | FV-PTC | Frozen | Negative | **65** | OTA | Frozen | Negative |
| **29** | FV-PTC | Frozen | Negative | **66** | OTA | Frozen | Negative |
| **30** | FV-PTC | Frozen | Negative | **67** | OTA | Frozen | Negative |
| **31** | FV-PTC | Frozen | Negative | **68** | OTA | Frozen | Negative |
| **32** | FV-PTC | Frozen | Negative | **69** | OTA | Frozen | Negative |
| **33** | PDTC | Frozen | Negative | **70** | OTA | Frozen | Negative |
| **34** | PDTC | FFPE | Negative | **71** | OTC | Frozen | Negative |
| **35** | FTC | Frozen | Negative | **72** | OTC | Frozen | Negative |
| **36** | FTC | Frozen | Negative | **73** | OTC | Frozen | Negative |
| **37** | Metastasis of FTC | FFPE | Negative | **74** | OTC | Frozen | Negative |
|  |  |  |  | **75** | OTC | Frozen | Negative |

**Table S1**
