# Supplementary material for: Identification of a Recurrent STRN/ALK Fusion in Thyroid Carcinomas
Source: PLoS One. 2014 Jan 27;9(1):e87170. doi: 10.1371/journal.pone.0087170 (PMC3903624; doi:10.1371/journal.pone.0087170)
Supplement: Table S2 — RT-PCR primers used. ALK and STRN forward and reverse primers are presented. Ex: exon, bp: base pair, FFPE: Formalin-fixed paraffin embedded, F: forward, R: reverse. (DOC) [file pone.0087170.s002.doc]

| **Primer Name** | **Primer sequence 5’🡪3’** | **Exon** | **Product size (bp)** | **Sample Series** |
| --- | --- | --- | --- | --- |
| **ALKex19F** | TGATCCTCTCTGTGGTGACCT | 19 |  | Both |
| **ALKex20R1** | TGCCAGCAAAGCAGTAGTTG | 20 | 199 |
| **ALKex20R2** | GGAGCTTGCTCAGCTTGTACTC | 20 | 147 |
| **STRNex2/3F** | GGAAAGAGCCAAATACCACAA | Junction ex2/3 | 110 | FFPE samples |
| **STRNex4R** | GTGGCTGCACTTCTGTTTCA | 4 |
| **STRNex4F** | GAAGTGCAGCCACAACAAAA | 4 | 159 |
| **STRNex5R** | CCTGTCCGTGACATCACTTG | 5 |
| **STRNex5F** | TGACAAAAATCAGGACTCAGTTG | 5 | 129 |
| **STRNex6R** | GCTGCACTTTCAAGGAATTTG | 6 |
| **STRNex6F** | GCAGATTTCAGTGATGAAGATG | 6 | 131 |
| **STRNex7R** | AGAGCTTCTTTTGTATCTCGATCT | 7 |
| **STRNex7F** | TTGGTTACATCAGAGGAAGGAGA | 7 | 104 |
| **STRNex8R** | TCCACATTCCAGGCTTCAG | 8 |
| **STRNex2/3F** | GGAAAGAGCCAAATACCACAA | Junction ex2/3 | 523 | Frozen samples |
| **STRNex7R** | AGAGCTTCTTTTGTATCTCGATCT | 7 |
| **STRNex6F** | GCAGATTTCAGTGATGAAGATG | 6 | 564 |
| **STRNex10R** | AAGGCCTGCTAGTTCTCCAA | 10 |
| **STRNex9F** | CATCTGTGGGTTCACCTTCC | 9 | 750 |
| **STRNex15R** | GATCCACAGAGGCAGGGATT | 15 |
| **STRNex14F** | CAGATGGCACTCTGCGTTTA | 14 | 517 |
| **STRNex18R** | TGGAAAGCTACATCATGAATCG | 18 |

**Table S2**
