# Supplementary material for: Identification of a Recurrent STRN/ALK Fusion in Thyroid Carcinomas
Source: PLoS One. 2014 Jan 27;9(1):e87170. doi: 10.1371/journal.pone.0087170 (PMC3903624; doi:10.1371/journal.pone.0087170)
Supplement: Table S3 — Genomic DNA primers used. ALK and STRN forward and reverse primers are presented. Ex: exon, bp: base pair. (DOC) [file pone.0087170.s003.doc]

| **Primer name** | **Primer sequence 5'-->3'** | **Product size (bp)** |
| --- | --- | --- |
| **ALKex19F** | TGATCCTCTCTGTGGTGACCT | 594 |
| **ALKgR1** | GGTTGGGACCACACTGAGTT |
| **ALKgF2** | TTTCTGTTCAGCCTGTGAGC | 637 |
| **ALKgR2** | CTGCCCTGTTTCCCTAACC |
| **ALKgF3** | TTTCAAAACCATTTTATGTTGGTG | 531 |
| **ALKgR3** | GCAAGTATAACCCCACGTGAA |
| **ALKgF4** | GGCCTTGCTGAAACTTCCTT | 521 |
| **ALKgR4** | GCATGGCTTGCAGCTCCT |
| **STRNgF1** | GGGACAGAATTGAATCAGGG | 629 |
| **STRNgR1** | TGTTTGATGGTATGAAGAAGTAGGA |
| **STRNgF2** | CATGTTTTAACAGCTGAACCATTT | 791 |
| **STRNgR2** | GTGACGTTGGGCAAGTTTTT |
| **STRNgF3** | TCCCGAGTAGCTGGGATTACT | 816 |
| **STRNgR3** | GCAGCCAGCCATGTTTACTT |
| **STRNgF4** | GGAGAATAGAAGCCTAGATGCAA | 700 |
| **STRNgR4** | GGGCATTGTTGTCTTATTCACT |
| **STRNgF5** | CCTAGTATCATTTCATGTATCTTTCCA | 670 |
| **STRNgR5** | CTCATTTGCCCAGGTTTCTG |
| **STRNgF6** | TACGTGGGCTGTAGTGTGGA | 699 |
| **STRNgR6** | CCTTCTGGAAGCCCTCTCTT |
| **STRNgF7** | GGTAGAAGAACGGAAACGTGA | 677 |
| **STRNgR7** | TCTTCCTGTTTACCATTAGCATTTT |
| **STRNgF8** | GATAGGATTATCTGCTGAGAGTGG | 660 |
| **STRNgR8** | CAGGCTCAATTACTCCCAATTC |
| **STRNgF9** | TGGCTAGAGGTCCCAGCTAA | 669 |
| **STRNgR9** | TGCAAATGGTCTAAATTTCTCAA |
| **STRNgF10** | TTTTTGATATTTCAGTCTCTCCTATTT | 646 |
| **STRNgR10** | CAAAAATTCATTACTTGCATTCCA |
| **STRNgF11** | TCCTTTGTGTAGATCTGTGTTTCC | 676 |
| **STRNgR11** | TGCCAAATTCCATGAAAACTA |
| **STRNgF12** | TTTCTTATGCTTGGGATTTATTGA | 579 |
| **STRNgR12** | CCTGGGCAACAGAGCTAGAG |
| **STRNgF13** | AAGAGCTATTTGACTTTGGGTTT | 694 |
| **STRNgR13** | TTAGCGTACGGAGGGAGAGA |
| **STRNgF14** | AAGAGGATAGGGTACATTCTTATTTGA | 598 |
| **STRNgR14** | CCCCCAAACTAAGGGAAAAC |
| **STRNgF15** | TGTTACCTGCACAGATCCTGA | 651 |
| **STRNgR15** | GGCAGAAGAATCGCTTGAAC |
| **STRNgF16** | AAATTCAACACTTACAATTGTGTTTC | 757 |
| **STRNgR16** | TTTGAATGAAAACCTCCTAAGCA |
| **STRNgF17** | GCTTGGAGATTGTACAGTCCAG | 697 |
| **STRNgR17** | TGATCGTGCCACTGTACTCC |
| **STRNgF18** | TGGCTCTTCTACCATCATTCC | 975 |
| **STRNgR18** | TGTGGCTGCACTTCTGTTTC |

**Table S3**
